# A Web-Based Clinical Decision Support Tool for Primary Health Care Management of Back Pain: Development and Mixed Methods Evaluation

**DOI:** 10.2196/resprot.3071

**Published:** 2014-04-02

**Authors:** David Peiris, Christopher Williams, Rachel Holbrook, Robyn Lindner, James Reeve, Anurina Das, Christopher Maher

**Affiliations:** ^1^The George Institute for Global HealthUniversity of SydneySydneyAustralia; ^2^Hunter Medical Research InstituteNewcastleAustralia; ^3^NPS MedicineWiseSydneyAustralia

**Keywords:** clinical decision support systems, back pain, primary care

## Abstract

**Background:**

Many patients with back pain do not receive health care in accordance with best practice recommendations. Implementation trials to address this issue have had limited success. Despite the known effectiveness of clinical decision support systems (CDSS), none of these are available for back pain management.

**Objective:**

The objective of our study was to develop a Web-based CDSS to support Australian general practitioners (GPs) to diagnose and manage back pain according to guidelines.

**Methods:**

Asking a panel of international experts to review recommendations for sixteen clinical vignettes validated the tool. It was then launched nationally as part of National Pain Week and promoted to GPs via a media release and clinic based visits. Following this, a mixed methods evaluation was conducted to determine tool feasibility, acceptability, and utility. The 12 month usage data were analyzed, and in-depth, semistructured interviews with 20 GPs were conducted to identify barriers and enablers to uptake.

**Results:**

The tool had acceptable face validity when reviewed by experts. Over a 12 month period there were 7125 website visits with 4503 (63.20%) unique users. Assuming most unique users are GPs, around one quarter of the country’s GPs may have used the tool at least once. Although usage was high, GP interviews highlighted the sometimes complex nature of management where the tool may not influence care. Conversely, several “touch-points”, whereby the tool may exert its influence, were identified, most notably patient engagement.

**Conclusions:**

A novel CDSS tool has the potential to assist with evidence-based management of back pain. A clinical trial is required to determine its impact on practitioner and patient outcomes.

##  Introduction

### Global Disease Burden From Back Pain

Back pain is the number one ranked disabling health condition worldwide [1]. Across 21 regions, the lowest ranking of burden was 4th of 291 diseases in both the Caribbean and Southern sub-Saharan Africa. This makes a compelling case for increased efforts to improve health care for this condition.

Back pain is best conceptualized as a chronic disease. Although symptoms usually improve quickly after onset, pain and disability frequently persist at low to moderate levels [2]. We found that nearly one third of the people with reported acute back pain still experienced pain one year later [3]. Around one quarter of those who recovered had a recurrence within one year [4], and the majority of those with a prolonged initial course of pain (> 3 months) reported persisting pain 12 months later [5]. Similar findings were noted in a UK study [6]. Thus, back pain is commonly a long term health condition with an unpredictable pattern of acute episodes, remission, and recurrence [7,8].

### Evidence-Practice Gaps in Management of Back Pain

Despite high health care expenditure for back pain, clear improvements in outcomes do not necessarily follow. In the United States, back pain expenditure increased by 65% (inflation adjusted) in the period 1997-2005, yet the health status for people with back pain fell [9]. Further, 16.7% of visits for back pain include inappropriate imaging tests, resulting in over $175 million per year of unnecessary expenditure [10]; the principal driver for this is that people with back pain are not receiving the care endorsed in guidelines. Our large survey of Australian primary health care providers revealed that few patients received the care recommended in the nationally endorsed guideline [11]. Less than 10% of the patients received appropriate analgesia, and just 20% were provided with appropriate advice. Additionally, over one quarter of the patients were referred for diagnostic imaging. Crucially, this pattern of care was essentially the same in the periods before and after the guideline was released in 2004 [11]. Similar evidence-practice gaps have been reported in other countries [12-14].

### Health Care Intervention Studies

Several health care intervention studies to improve back pain management have resulted in minimal or no improvements in mainly process outcome measures [15-20]. Clinical decision support systems (CDSS) are one of the most promising interventions to improve uptake of guideline recommendations. In two recent systematic reviews, around two thirds of CDSS trials demonstrated improvements [21,22]. In the broader eHealth arena, there is now growing evidence that interactive Internet and mobile interventions improve outcomes from chronic conditions. A Cochrane review of 124 studies concluded that computer based “Interactive Health Communication Applications” could improve cognitive and social support outcomes in patients with chronic conditions [23]. Despite this, we are not aware of any CDSS that have been trialed for back pain management.

In this paper, we outline the development of a Web-based CDSS for back pain management. We also discuss the development and validation of an algorithm, in addition to a feasibility study examining the acceptability and utility of the tool following national deployment for use by Australian general practitioners (GP). Our objective was to determine whether the tool had the potential to support GPs to diagnose and manage back pain in accordance with guideline recommendations, and to identify barriers and enablers to uptake. The study was designed to inform a future large scale trial evaluation.

## Methods

### Tool Development

**Figure 1:**
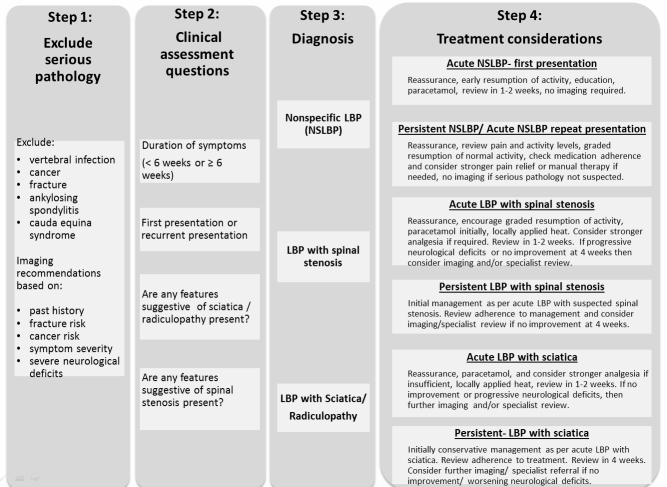
Decision support flowchart for management of low back pain (LBP). Nonspecific LBP (NSLBP).

First, our recent systematic search of relevant guidelines was drawn on to inform development of the decision support algorithm [24]. In this review, we searched for guidelines in MEDLINE, PEDro, the US Agency for Health Care Research and Quality National Guideline Clearinghouse, and the UK National Institute for Health and Clinical Excellence, and used backward and forward citation tracking, retrieving 15 eligible guidelines. A key finding from the review was that there was a high level of consistency in recommendations from all 15 guidelines. These recommendations were converted to plain language rules, hardcoded into an algorithm, and a prototype tool was developed. Figure 1 illustrates the key variables in the algorithm and treatment recommendations provided.

Second, the algorithm was validated as follows. There were 16 hypothetical cases representing all permutations of the algorithm that were entered into the tool to generate management recommendations. The output was compared to an expert's clinical decisions for each case based on their interpretation of the guidelines. Additionally, a panel of four experts who developed the UK, US, and Australian guidelines [25-27] reviewed the prototype and commented on the recommendations. Any misinterpretations were reviewed and inconsistencies resolved by a consensus process.

**Figure 2:**
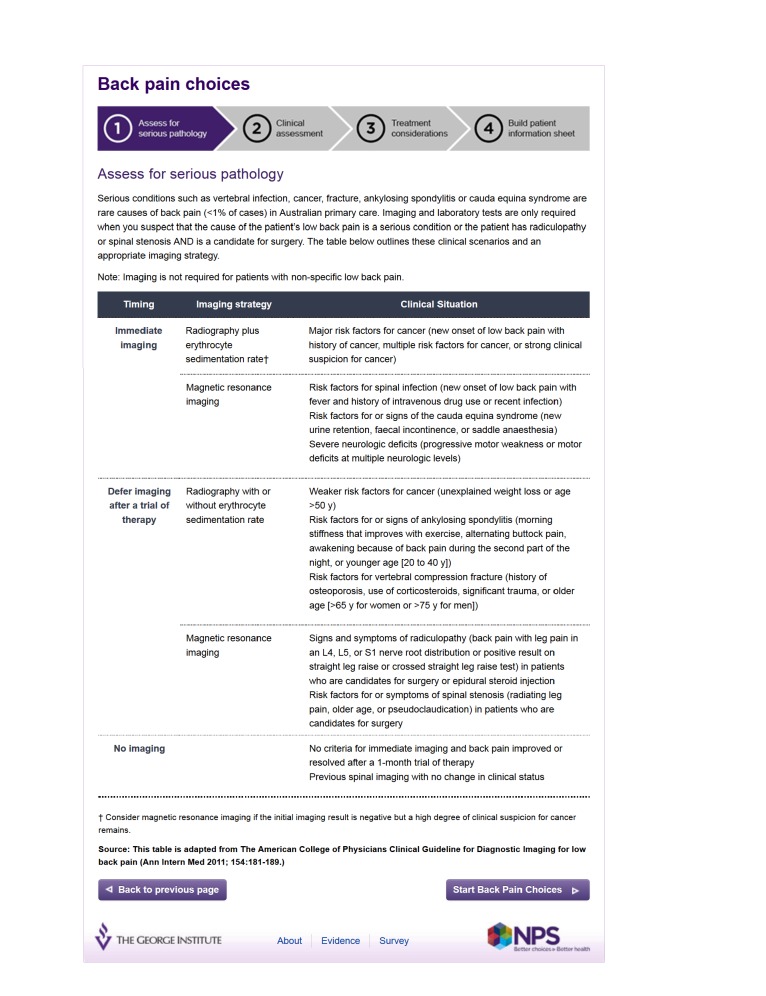
Screenshot Step 1 - Exclude serious pathology.

**Figure 3:**
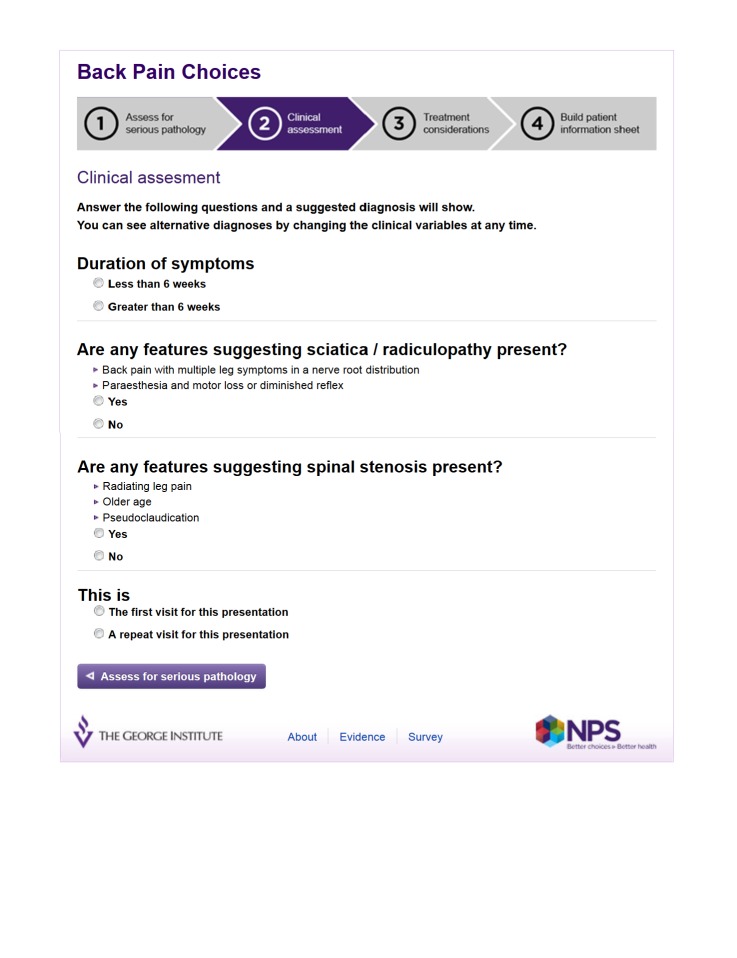
Screenshot Step 2 - Clinical assessment.

**Figure 4:**
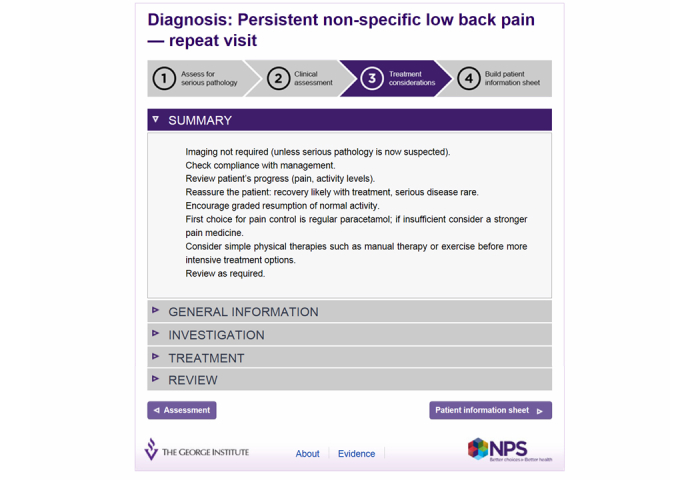
Screenshot Step 3 – Treatment considerations.

**Figure 5:**
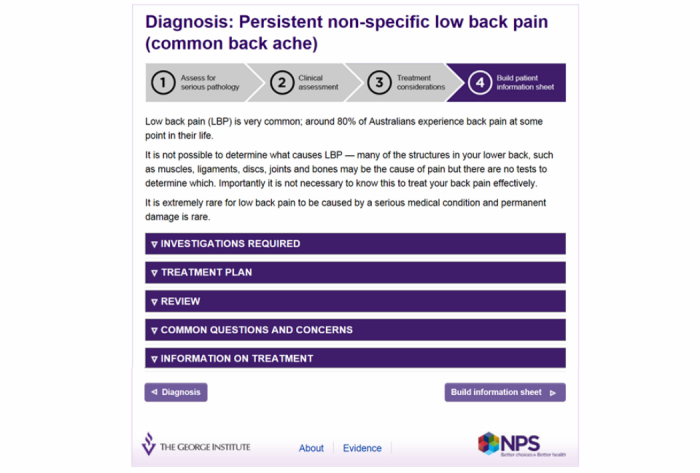
Screenshot Step 4 – Build information sheet.

Third, a user interface was designed, resulting in a four step process of excluding serious pathology, clinical assessment, establishing treatment options, and building a personalized, patient information sheet (Figures 2-5 show this interface). Technical user acceptance testing was conducted to ensure the tool functioned as specified.

### Implementation

The tool was published on the Internet in June 2012, accompanied by a media release issued to coincide with Australian National Pain Week [28]. The tool was subsequently mentioned in several leading GP focused periodicals, the Royal Australian College of General Practitioners electronic newsletter, sent to over 20,000 GPs, the Australian Medicare Local Alliance newsletter, and circulated to all 61 Medicare Locals, which are government funded, middle tier primary health care organizations. The tool was also directly promoted via a NPS MedicineWise “visiting topic”, in which physiotherapist educators conducted small clinic based workshops to support GPs in improved health care quality.

Following this, a convenience sample of twenty GPs in the Sydney area were invited to use the tool over a four week period in order to provide further insights into its utility, barriers, and enablers to uptake.

### Evaluation

A mixed methods evaluation was conducted of the tool. Usage data were collected using Google Analytics over a 12 month period. Data included visit frequency, number of unique visitors, number of page views, and average visit duration. The qualitative component involved in-depth, semistructured interviews with all 20 GPs who had trialed the tool. A GP interviewer who was not involved in tool development conducted the semistructured interviews (see Multimedia Appendix 1 for the interview guide). The interviews covered four key domains: (1) opinions on back pain management, (2) a “live walk-through” of the website, (3) clinical and patient perspectives on utility, and (4) recommendations for improvements. The interviews were digitally recorded, transcribed, and imported for analysis into NVivo 10 (QSR International). An inductive approach was taken, with thematic content analysis conducted contemporaneously with data collection. A preliminary coding framework was collaboratively derived from the analysis of four interviews, discussed by the broader project team, and revised accordingly. There were two researchers that then independently coded the remaining interviews (eight each), and discussed new themes not previously identified. Saturation of themes occurred after around 15 interviews. At the end of the interview coding, final findings were again discussed by the project team and key themes were further refined.

The University of Sydney Human Research Ethics Committee approved the study.

## Results

### Website Usage Data

**Figure 6:**
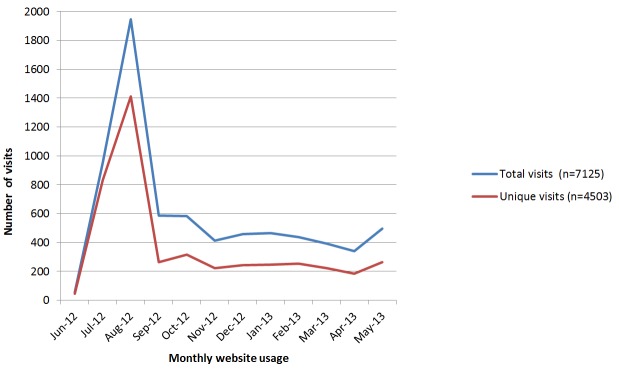
Usage patterns of the decision support tool for June 2012-May 2013.

Figure 6 shows the usage data from June 2012 to May 2013 of the tool. The peak in early August 2012 coincides with the media release; subsequent to this usage, patterns remained relatively constant. The average time spent per visit was just over four minutes and the average number of pages viewed per visit was six pages. Almost all visitors clicked through to at least the second page of the back pain assessment.

### Qualitative Evaluation

Table 1 outlines the characteristics of the GPs interviewed that had trialed use of the tool.

**Table 1:** GP and practice characteristics.

Characteristics			Total=20, n (%)
**GP characteristics**			
	**Age, years** ^a^		
		<50	8 (40)
		50-59	8 (40)
		60+	3 (15)
	Male		13 (65)
	English primary language spoken at home		14 (70)
	Australian university graduate		15 (75)
	Vocationally registered		20 (100)
	**Number of sessions worked per week**		
		2-5	4 (20)
		>5	16 (80)
	Participates in research often or very often		6 (30)
	Conducts own research		8 (40)
	Daily use of Internet or email for work purposes		20 (100)
**Practice characteristics**			
	**Number of patients registered**		
		1001-3000	1 (5)
		3001-5000	7 (35)
		>5000	12 (60)
	**Number of doctors**		
		1-5	9 (45)
		6-10	9 (45)
		>10	2 (10)
	**Number of nurses**		
		0	2 (10)
		1-3	15 (75)
		>3	3 (15)
	Practices with a practice manager		17 (85)
	GP registrar placement		4 (20)

^a^One response missing

### Identified Themes

**Figure 7:**
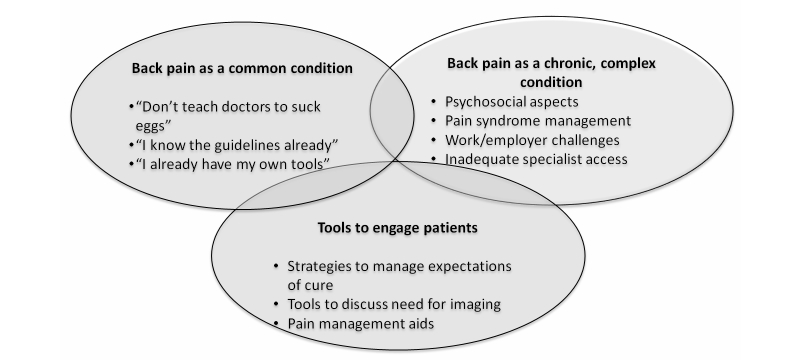
Thematic domains from in-depth interviews with twenty GPs.

There were three interrelated themes on the management of back pain and the role of tools that were identified. An additional theme, focused on the functional aspects of the tool, also yielded important information on recommendations for improvement. Figure 7 shows the key thematic domains and related subthemes.

### Theme 1: Low Back Pain Is a Common Condition

Our motivation for developing the CDSS tool was to improve adherence to guideline recommendations. Many of the GPs interviewed, however, considered back pain diagnosis and management to be common and relatively straightforward. It was viewed as integral to being a competent practitioner, and, therefore, the notion that GPs might need a tool to assist them seemed to indicate that “something was wrong” with their skills.

You can’t have a tool for every presentation in general practice...this is something that should be up there (pointing to his head) rather than “Oh wait a second, let me use this tool...”

GP 3

Similarly, one GP was worried that the use of such a tool during a consultation would make him look “a bit strange” in the eyes of his patient, while another felt it was condescending and that he did not need “a step-by-step approach on how to suck an egg.” These accounts highlight the tension between a perceived, sound knowledge of best practice and the published data demonstrating large evidence-practice gaps.

### Theme 2: Low Back Pain Is a Complex, Chronic Condition

Juxtaposed with the notion that back pain is common, was a more nuanced appreciation that it may have a complex, chronic course. This course can be characterized by relapse and setbacks, psychosocial and work related repercussions, and fragmented health care experiences. Most GPs acknowledged that under these circumstances back pain management was challenging and required considerable effort to ensure satisfactory health care. These challenges are particularly pronounced with work related back pain, where health care costs are subsidized in Australia by the government worker’s compensation schemes.

For one patient injured at work...he seems to be using it to get back at his employer...It also looks as though his relationship (with his partner) is going to break down...and he fosters a sense of entitlement through his back pain...

GP 2

In such circumstances, GPs may be more likely to conduct imaging tests or prescribe complex pain relief medications, while cognizant that these are not routinely indicated.

I tried to get (him) managing the pain, but he was also a very anxious man. He went to my colleague, had a CT scan, which then showed something he fixated on. It’s been years to get him to a point where he’s not obsessing about every twinge in his back.

GP14

### Theme 3: Tools to Engage Patients

We came to appreciate that the tool was situated amid highly varied clinical contexts, ranging from where GPs required minimal additional support, through to complex situations where new resources were desirable. This helps to explain why at times the tool was seen as superfluous, and at other times it had the potential to assist with management dilemmas.

The tool was perceived to be the most useful in situations where patient reassurance and avoidance of complex tests or medicines were recommended. The GPs used the tool as a source of authoritative advice to support their recommendations. The patient information sheet was a particularly useful component, as it synthesized the key messages that would otherwise take some time to explain.

Well, I suppose we tended to make the patients feel happy if they want x-rays...Now at least we can say, “This is the evidence. You’ve got to wait for a while...”

GP 3

Similarly, in the context of a work related injury, one GP used the tool to communicate with the patient’s employer.

I used the tool for the employer because the patient refused to go back to work...So I copied a few things from the tool and emailed it to the employer and they were quite happy.

GP 5

### Theme 4: Recommendations to Improve Tool Content

While the tool had the potential to be of value, some GPs found that it was not sufficiently dynamic to be of use in the long term. Once the core components became clear, there was perceived to be little variation in the content. This was not necessarily an adverse finding, as many GPs felt that over time the recommendations would be “incorporated as part of their normal skills.” Some GPs, however, wanted more detailed advice for complex pain management and indications for referral, whereas others wanted more autonomy in customizing the patient information.

The main influence to future tool uptake appeared to be integration into routine workflow. Integration with clinical software systems and the ability to rapidly navigate to the parts of immediate interest were frequently recommended by the GPs. They were also concerned with the growing number of tools that compete for their attention. Many described a process by which they establish their own “tool box” of resources, often compiled over many years. Tool developers therefore need to make new tools that complement rather than compete with existing tools.

##  Discussion

### Principal Results

This study examined the development, uptake, and acceptability of an Australian GP Web-based CDSS for the management of back pain. There were three key findings that were observed. First, the algorithm underpinning the tool appeared to have acceptable face validity when reviewed by experts in the field and when implemented among GPs. This reflects consistency in guideline recommendations over several years. Second, we identified an effective implementation mechanism for rapid dissemination of the tool, drawing on a multi-pronged approach of media releases in target periodicals, alignment with National Pain Week, and promotion via an existing national network of facilitators who conduct face-to-face GP visits. Assuming that most unique visits were GPs, and that there are 20,360 GPs in Australia [29], then, over a 12 month period, around one quarter of the GPs in the country used the tool. Moreover, the ratio of new to returning users has remained constant, indicating the tool has not yet reached full market saturation. Third, and perhaps most importantly, the qualitative component revealed the complexities of managing low back pain and the “touch-points”, whereby a CDSS tool may exert its influence.

### Limitations

This study was exploratory in nature and did not assess the clinical effectiveness of the tool. The focus was to look at usability factors, identify enhancements needed, and further refine the tool. Further, although the usage data are useful, we were unable to analyze use by provider type, and to examine in more detail which parts of the tool were most/least popular. A final limitation was that we did not examine the perspectives of patients and other health care providers, such as physiotherapists and specialists.

### Comparison With Prior Work

To our knowledge, this is the first CDSS for back pain to be evaluated. Although uptake and general satisfaction was encouraging, ongoing usage patterns are likely to be highly variable. Our findings resonate with our recent study of a cardiovascular CDSS tool and outline that GPs adopt guideline recommendations judiciously, depending on multiple social and environmental factors [30,31]. This may partly explain why interventions, including Internet interventions to improve back pain management, have thus far yielded small effects at best [32,33]. The notion that GPs establish their own “tool box” to conduct their work is useful in understanding how a new tool will be perceived. For common conditions like back pain, this compendium of existing tools tends to be well established, and, consequently, a tool that covers only the basics is likely to be less effective. Conversely, GPs clearly experience challenges in effectively managing back pain for their patients. If a CDSS tool can support GPs in these domains, especially complex pain management, psychosocial impact, and work related issues, then such a tool is likely to be concordant with their needs. Tool customization was of particular importance to the GPs. In a model akin to a mobile “app store”, GPs could select and modify their preferred “apps” on the basis of personal interest, user feedback, or endorsement by trusted organizations.

### Conclusions

Despite the existence of clear and consistent recommendations from national and international guidelines for the management of back pain, large evidence-practice gaps exist. Our findings suggest that decision support tools have the potential to serve as an effective dissemination mechanism for the implementation of guidelines. This study represents the initial stage of a research translation program to improve health care quality for back pain. Several tool features were identified to be useful, but, importantly, a number of contextual factors affecting how back pain is managed were also noted. These factors may require additional tools with a different focus. Further work with care providers and consumers is planned to develop and test these enhancements. The system will then be assessed for its effectiveness in improving health care for the world’s most disabling health condition.

## Multimedia Appendix 1

Interview guide.

## Abbreviations

CDSS: clinical decision support system
GP: general practitioner
