# Supplementary material for: A Web-Based Clinical Decision Support Tool for Primary Health Care Management of Back Pain: Development and Mixed Methods Evaluation
Source: JMIR Res Protoc. 2014 Apr 2;3(2):e17. doi: 10.2196/resprot.3071 (PMC4004153; doi:10.2196/resprot.3071)
Supplement: Supplementary file 1 [file resprot_v3i1e17_app1.pdf]

INTERVIEW GUIDE- BACK PAIN CHOICES

I'd like to ask you some questions about Back Pain Choices based upon your recent experience using the tool. There are no wrong or right answers and we are very much interested in your feedback and suggestions for improvement.

Question 1: What do you consider are the key issues for GPs in managing low back pain?

Probes:

- How confident do you feel managing back pain? Why?
- Can you describe an experience where you found managing back pain very challenging? Why?
- Do you consult any guidelines for the management of back pain?
    - If so which ones?
    - How easy are these guidelines to use?
- Is there a need for tools like Back Pain Choices?

Question 2: I'd like you to open up the tool now so we can discuss some of the content…What was your overall impression of the Back Pain Choices tool?

Probes:

- Did you find it easy to navigate to the tool website?
- Once you entered the website how easy was it to navigate?
    - What did you find useful?
    - What information was not helpful?
    - Was there anything confusing about the tool?
- How satisfied were you with the management recommendations?
    - If not satisfied how could it have been done differently?

Question 3: What were your patients' impressions of using the tool?

Probes:

- Did you use the tool with your patients during the consultation?
    - Why/ Why not?
- Were there any features of the tool that patients found useful?
    - What kinds of things did your patients say about the tool?
- Did the tool have any impact on how management decisions were made?
- Did you generate a patient information sheet for any of your patients?
    - If not why not?
    - If yes how useful was this sheet?
- How do you think the tool could be improved for use with patients?

Question 4: What advice do you have for us in implementing Back Pain Choices into routine health care?

Probes:

- How often do you think you would use this tool in your everyday practice?
    - Would you use routinely for every patient with back pain?
    - Would you use it for patients with recurrent presentations of acute back pain/ chronic back pain?
    - Would you only use in cases where you were unsure?"
- If we integrate this tool into GP software
    - What do you see as its potential benefits?
    - Would you anticipate any disadvantages?
    - What barriers do you think we would face?
